# Recent Advances on Electrospun Nanomaterials for Zinc–Air Batteries

**DOI:** 10.1002/smsc.202100010

**Published:** 2021-05-25

**Authors:** Chenfeng Xia, Yansong Zhou, Chaohui He, Abdoulkader Ibro Douka, Wei Guo, Kai Qi, Bao Yu Xia

**Affiliations:** ^1^ Key Laboratory of Material Chemistry for Energy Conversion and Storage (Ministry of Education) Hubei Key Laboratory of Material Chemistry and Service Failure Hubei Engineering Research Center for Biomaterials and Medical Protective Materials Wuhan National Laboratory for Optoelectronics School of Chemistry and Chemical Engineering Huazhong University of Science and Technology (HUST) 1037 Luoyu Rd Wuhan 430074 China

**Keywords:** electrocatalysts, electrospinning, membranes, nanofibers, zinc–air batteries

## Abstract

Zinc–air batteries have received increasing attention in energy storage and conversion technologies. However, several challenges still emerge in the development of high‐level zinc–air batteries. In this regard, electrospun materials with unique nanostructures and characteristics are applied in the high‐performance zinc–air batteries. This work reviews recent progress of electrospun technologies for their diverse application in novel zinc anodes, separators, and air cathodes in zinc–air batteries. After a brief introduction, the fundamental principle and technological parameter of electrospinning technology are discussed, and then multifunctional electrospun nanofiber materials including their fabrication procedures, structures, and electrochemical properties are reviewed in the application of zinc–air batteries. Finally, urgent challenges and opportunities are comprehensively proposed for the electrospun nanomaterials in zinc–air batteries. This work intends to offer readable report for electrospun technology toward their application in zinc–air batteries, which inspires more fabrication approaches and material innovations in energy conversion and storage technologies.

## Graphical abstract

Electrospinning technology and its fabricated nanofibers for applications in the air cathodes, separators, electrolytes, and zinc anodes in zinc–air batteries are comprehensively reviewed. This work offers insightful understanding in future development of electrospinning technologies and zinc–air batteries.

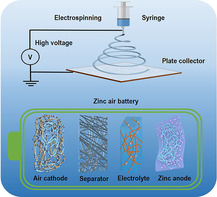

## 1 Introduction

Metal–air batteries such as zinc–air batteries (ZABs), lithium–air batteries, and magnesium–air batteries are considered as the promising energy storage and conversion devices. Due to their advantages of low cost, high specific energy, stable discharge voltage, and environmentally benign, these devices are suitable for responding the fast‐growing demands in mobile communication, emergency storage, electric vehicles, and large‐scale energy storage.^[^ ^1^, ^2^, ^3^, ^4^ ^]^ Especially, ZABs are regarded as one of the most potential metal–air batteries due to the natural profusion of zinc, high‐volume ratio (theoretical specific energy density 1218 W h kg^−1^), and stable chemical properties.^[^ ^5^, ^6^ ^]^ Similar to the working principle of fuel cells,^[^ ^7^, ^8^ ^]^ the energy is released through the oxidation of zinc at the anode and the reduction of oxygen at the cathode in ZABs. The zinc anode should be uniformly oxidized and reduced during the discharge and charge process whereas the oxygen reduction and evolution reaction (ORR and OER) at the air cathode should be speedily for the energy conversion efficiency and stably proceed for the long‐life service. Therefore, an appropriate synergy between zinc anode and air cathode is needed to achieve the high‐performance ZAB.^[^ ^9^, ^10^, ^11^ ^]^ Bold research trials with respect to the design and synthetic techniques for diverse components of zinc anodes, separators, and air catalysts/cathodes have been developed to achieve high‐performance ZABs.^[^ ^12^, ^13^, ^14^, ^15^ ^]^

Electrospinning technology can easily control the spinning process using the simple device to produce submicron composites and ceramic nanofibers, which can be applied in various fields of renewable materials, energy storage and conversion equipment, gas filtration, pollution purification, and wound dressings.^[^ ^16^, ^17^, ^18^, ^19^, ^20^ ^]^ These electrospun nanofiber materials with unique porous structures, large specific surface area, sufficient active sites, superior conductivity, and prominent flexibility can also be widely applied in various components of ZABs.^[^ ^21^, ^22^, ^23^ ^]^ The electrospun nanofiber materials can be attached to electrode substrates as the air catalysts or be directly functioned as the free‐standing integrated air cathodes for the ZABs.^[^ ^24^, ^25^ ^]^ However, it is worthy to note the pristine nanofibers prepared by electrospinning may not effectively and adequately meet the requirements for the high‐performance ZABs. Therefore, further functionalizations of electrospinning precursors as well as the post‐treatment of as‐prepared electrospun nanofibers are usually performed to improve their performances.^[^ ^26^, ^27^, ^28^ ^]^ The electrospun nanofibers prepared by the multifunctional precursors can achieve higher specific surface areas, larger porosity, adjustable chemical composition, and controllable fiber assembly structures. In addition, the modified electrospun nanofibers possess excellent catalytic properties and improved stability compared with their pristine counterparts.^[^ ^29^, ^30^ ^]^

In the past few years, some reviews have summarized the applications of electrospinning technology in other applications, it is necessary and yet significant to comprehensively discuss these advanced electrospun technology for the fabrication of diverse components of ZABs.^[^ ^31^, ^32^ ^]^ In this contribution, we will provide a readable overview on electrospinning technology for their relative application in ZABs. This work begins with a brief background of electrospinning technology and its working principles, the specific characteristics of electrospinning technology are then discussed in detail. Moreover, recent research progress in diverse ZABs components achieved by the electrospinning technology is summarized, including their fabrication procedures, morphologies, structures, and electrochemical properties. Finally, the challenges, prospects, and opportunities of electrospinning technology for improving the battery performance and expediting the practical applications of high‐performance ZABs are presented. It is hoped that this review would provide some useful guidelines and inspirations for developing stable, low‐cost, and high‐efficiency ZABs, which would also inspire the development of sustainable energy conversion and storage technologies.

## 2 Electrospinning Technology

### 2.1 Electrospinning Principle

**Figure 1:**
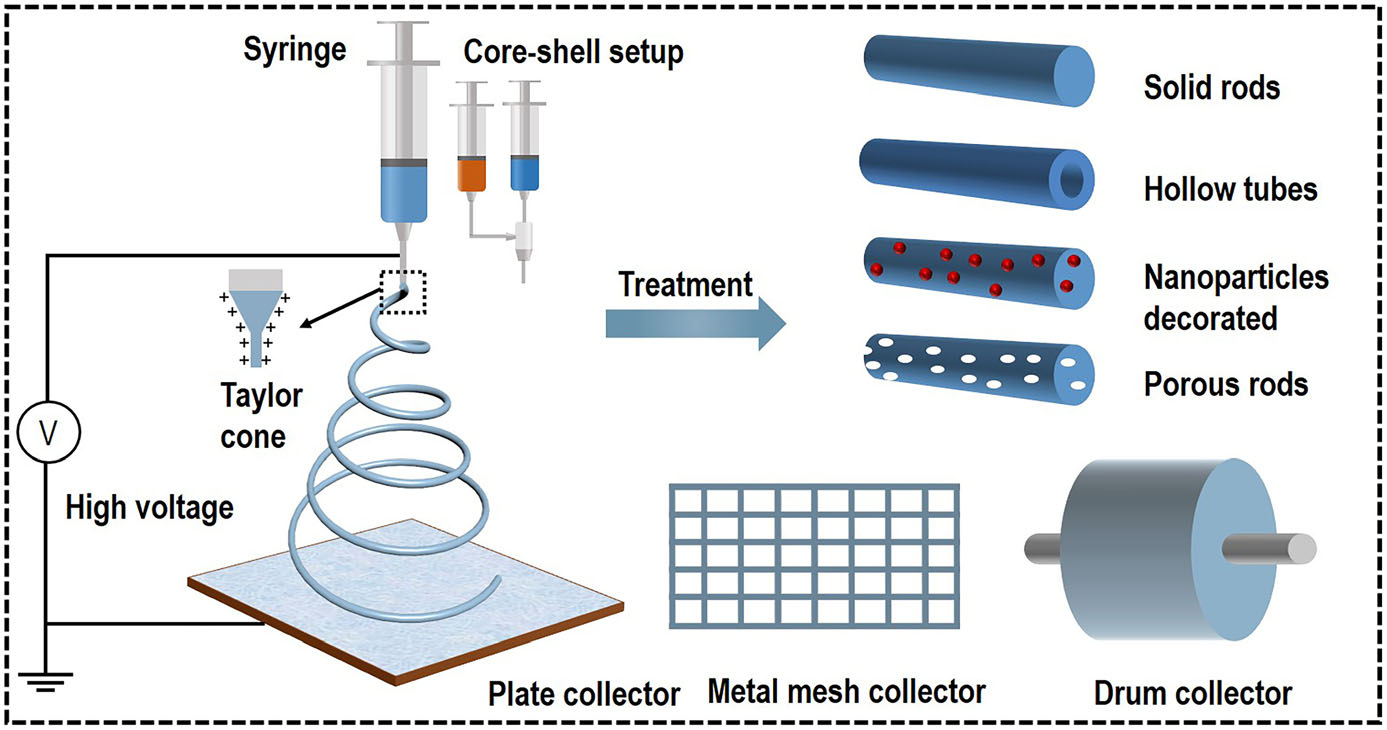
Schematic illustration of electrospinning and their fabricated nanostructures.

Electrospinning has a long history and a wide application in our daily life.^[^ ^33^, ^34^, ^35^, ^36^, ^37^, ^38^ ^]^ Its basic device and spinning process can mainly be divided into three parts, as shown in **Figure** **1**. The first part is the high‐voltage device, which is the key to the spinning of nanofibers. The second part is the syringe and corresponding propulsive injection device. And the third part is the conductive collection substrate, drum, and metal mesh. The finally produced electrospun nanofibers usually demonstrate four types of structure, including the solid rods, hollow tubes, nanoparticles decorated, and porous rods, which can represent most of the electrospun nanofibers and derived structures. These structures, as basic types, provide a tunable platform for further modification and functionalization besides their own special characters, such as the large specific surface area, sufficient exposure of active sites, and low conductivity.

### 2.2 Precursor Solution

**Figure 2:**
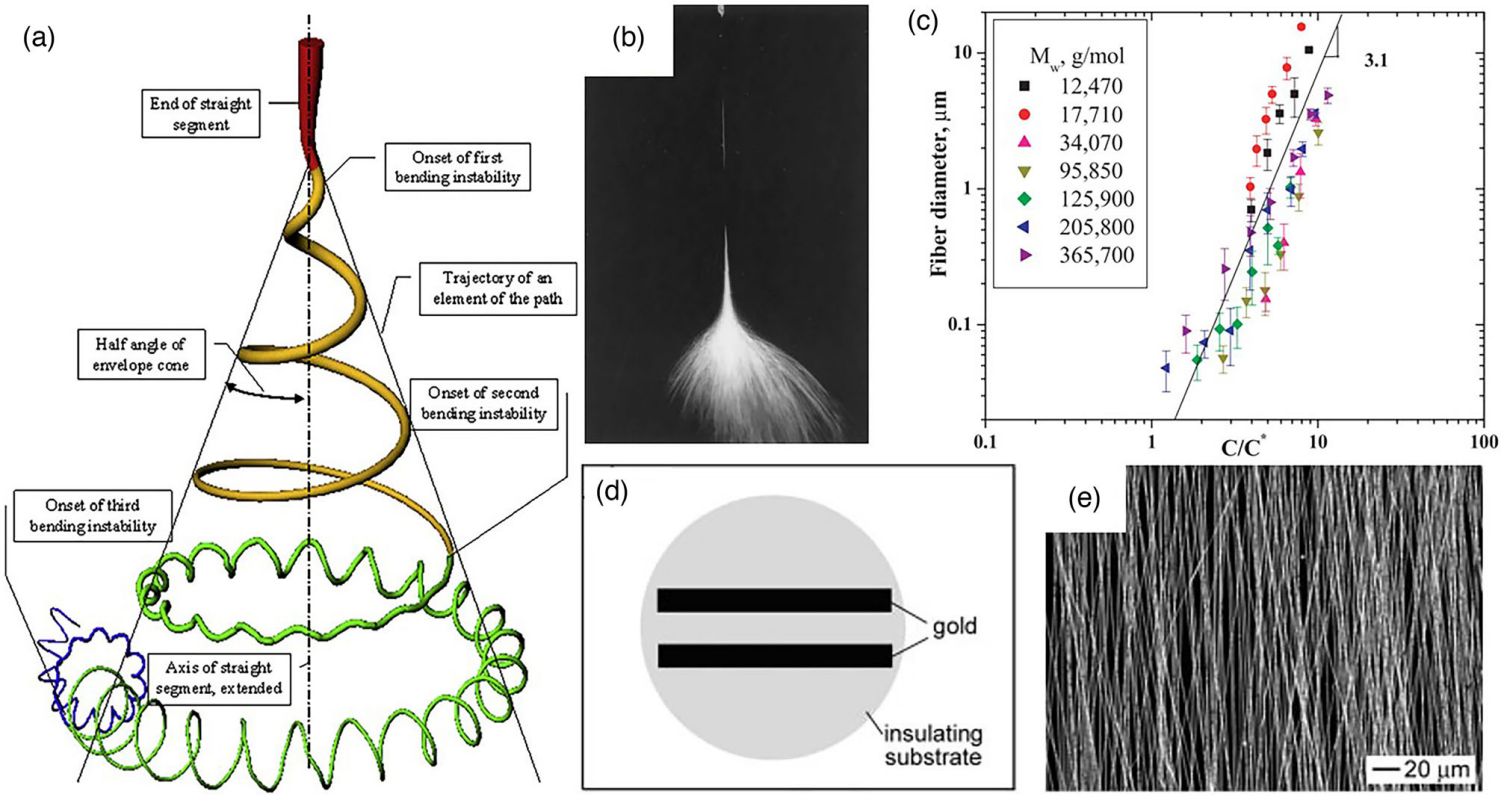
a) Schematic illustration of an electrospun jet. Reproduced with permission.^[^ ^39^ ^]^ Copyright 2006, American Chemical Society. b) Typical high‐speed photography of electrospun fiber whipping. Reproduced with permission.^[^ ^40^ ^]^ Copyright 2001, Elsevier. c) The relationship between different power input and resultant PMMA fibers. Reproduced with permission.^[^ ^41^ ^]^ Copyright 2005, Elsevier. d) Diagram of a parallel electrode collector and e) scanning electron microscopy (SEM) image of highly aligned electrospun nanofibers. Reproduced with permission.^[^ ^43^ ^]^ Copyright 2004, Wiley‐VCH.

A more specific spinning process is shown in **Figure** **2**a,b.^[^ ^39^, ^40^ ^]^ Under the force of gravity or external force like electrostatic repulsion, the electrospun precursor solution in the syringe will form conical droplets (Taylor cone) at the tip of the needle. When the accumulated charge on the droplet surface is beyond the surface tension, the precursor solution will flow out from the syringe to become a jet in the air, then the solvent volatilizes gradually, resulting in the formation of final nanofibers. These nanofibers will drop irregularly in the environment, and finally the macroscopic film can be obtained on the conductive collection substrate. The real way of the ejected nanofibers in the preparation process is shown in Figure 2b. Meanwhile, to obtain diversified and functional electrospun nanofibers, many condition variables can be used to control and regulate the electrospinning technology. The most important internal factor should be the precursors, since the combination of different polymers and solvents will change the boiling point of the solvent and solution viscosity coefficient, which ultimately determine the feasibility of electrospinning.^[^ ^18^ ^]^ For example, the inappropriate molecular weight will hamper the solution and nanofibers preparation, which will affect the amount of polymer chain entanglement in the solution and change the bond with the polymers. Recently, seven linear poly(methylmethacrylate) (PMMA) polymers with molecular weights (*M*_w_) ranging from 12 470 to 365 700 showed that with the gradual increase in molecular weight,^[^ ^41^ ^]^ the numbers of bead nodes and droplets on the electrospun nanofibers gradually decreased, and the diameter of the electrospun nanofibers increased (Figure 2c). For the soluble materials, the rational selection of solvent is of great importance, and the amount of dosage should be controlled.^[^ ^19^ ^]^ The solvent itself should have a good volatility and decent conductivity, which can not only accumulate sufficient charge in the electric field but also overcome the surface tension to obtain the desired nanofibers. For instance, polystyrene (PS) can be prepared by using carbon disulfide (CS_2_), toluene, and tetrahydrofuran/dimethylformamide (THF/DMF) mixture as different solvents.^[^ ^42^ ^]^ Due to the selection of different solvents, the obtained surface morphology and diameter distribution of nanofibers are also varied, suggesting the solvent volatility plays a pivotal role in the electrospinning process.

### 2.3 Process Parameter

In the actual preparation process, it is important to carefully consider the external parameters including applied voltage, injection rate, and the distance between the needle and substrate.^[^ ^22^, ^23^ ^]^ For instance, the presence of voltage is the premise to form nanofibers. A higher working voltage can increase the charge accumulation on the surface of the needle drop and the acceleration of jet, making more jets squeezed out at the same time, resulting in a smaller fiber diameter. However, excessive voltage will shorten the squeezing time from the jet to reach the substrate, and the solvent will not be completely volatilized, resulting in a spinning failure. A smaller voltage will also prevent the droplet from becoming a jet, making the fiber diameter thicker or even failure of the electrospinning process. Therefore, the voltage range needs to be appropriately adjusted to find the optimal value. Under the impulse of the pump, the solution can flow out of the syringe into a jet with a fixed injection rate at a certain speed, which is similar to the function principles of changing the voltage. The flow rate of the solution has a minimum value to maintain the nanofibers formation. The diameter and morphology of the nanofibers can also be changed by adjusting the distance between the needle and the substrate, which are mainly used to affect the volatilization process of the solvent in the jet and the degree of fiber stretching. As the distance increases, the diameter of the nanofibers will become smaller, which also corresponds to the changes in voltage and injection rate. A minimum distance of solution should also be noticed during the electrospinning procedure. If the distance is too small, the solvent would not evaporate immediately and the nanofibers cannot be fully stretched.

### 2.4 Collection Substrate

The electrospun nanofibers formed through a series of steps need to be collected by a specific conductive collection substrate. Indeed, different types of substrates will have various effects and influences on the nanofibers.^[^ ^37^, ^38^ ^]^ The most common conductive collection substrate is the flat metallic plate collector, similar to the stainless‐steel plate and metal mesh. Although the device is simple and low cost, the obtained nanofibers are often randomly oriented, and the morphological structure is not easily controlled. As an evolution, the drum‐type collection substrate can adjust the collection speed. Under high‐speed rotation, uniform nanofibers with high orientation can be obtained, but it is often difficult to obtain a thicker fiber membrane. In addition, the parallel electrodes can act as the substrates to collect straightened nanofibers, modifying two silicon strip electrodes on silicon strips and placing the two electrodes in parallel with a certain gap (Figure 2d,e).^[^ ^43^ ^]^ Attractively, the electrospun nanofibers will be straightened and arranged between the electrodes under the electric field force, and finally form the aligned fibers. This novel substrate design provides an efficient method for obtaining nanofibers with specific patterns. In general, due to the inherent properties of the precursor solution and the external experimental parameters, these nanofibers can possess various special morphologies. The as‐obtained nanofibers can be further treated by pyrolysis for a variety of unexpected properties.

## 3 Electrospinning in ZABs

**Figure 3:**
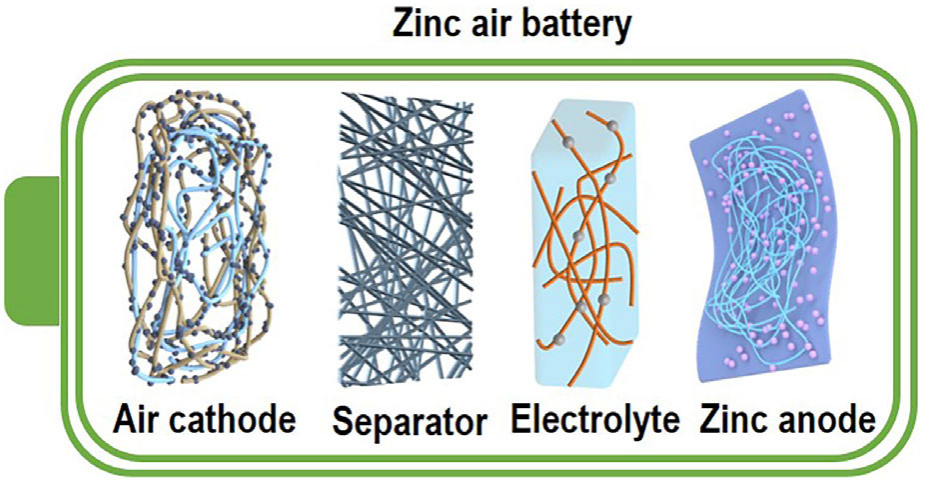
Schematic diagram of electrospun nanostructures in ZABs.

**Figure** **3** shows a typical schematic diagram of an aqueous ZAB, which is mainly composed of an air cathode with a catalyst layer, an alkaline electrolyte, a separator, and a zinc anode. These parts synergistically determine the battery performance, such as stability, safety, specific capacity, and rate performance.^[^ ^44^, ^45^ ^]^ Currently, many technologies have been developed to improve these parts within ZAB. Among them, the electrospinning technology as a novel, facile, and effective synthetic technology has been widely adopted in the component fabrication of ZABs.

### 3.1 Zinc Anode

**Figure 4:**
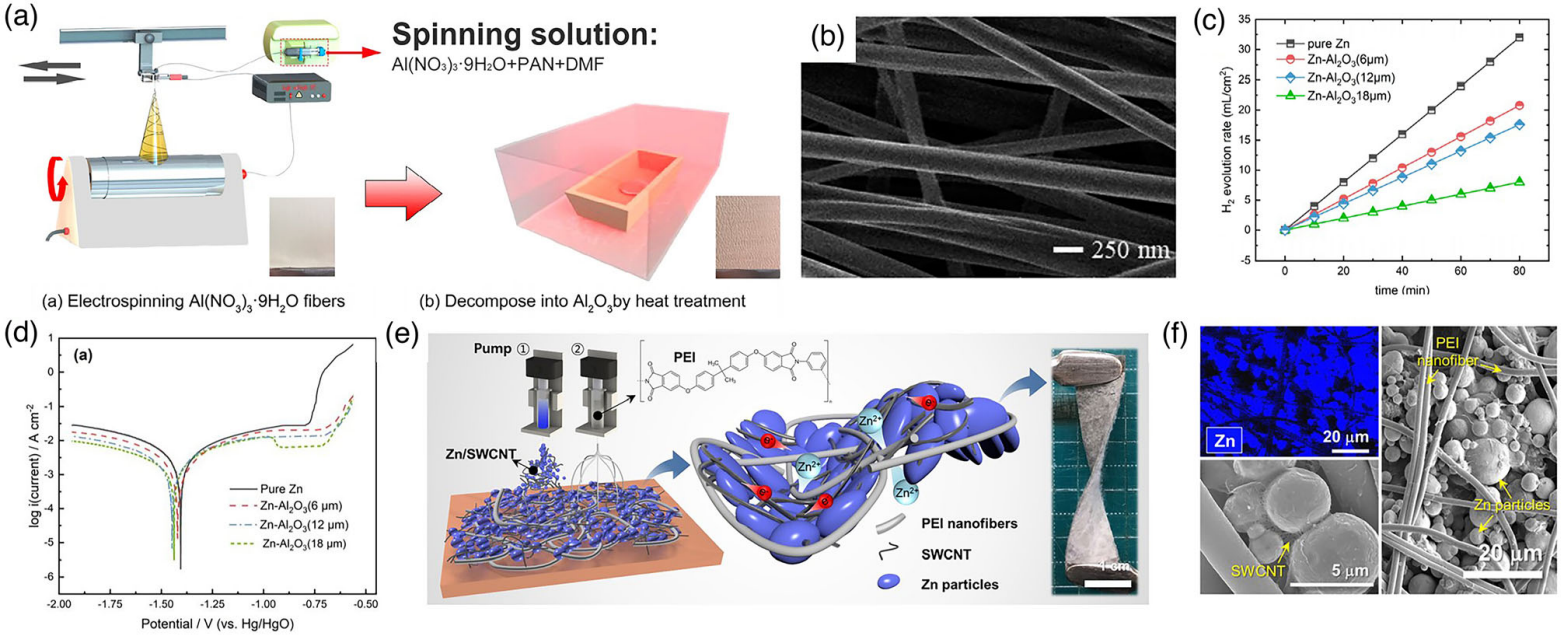
a) Fabrication process and b) SEM images of Al_2_O_3_ coatings. c) Hydrogen evolution rates and d) polarization curves of Zn anodes with different thicknesses of Al_2_O_3_. a–d) Reproduced under the terms of the CC‐BY 4.0 license.^[^ ^54^ ^]^ Copyright 2019, The Authors, published by MDPI. e) Synthetic process and f) SEM images of HM Zn anode. e,f) Reproduced with permission.^[^ ^55^ ^]^ Copyright 2018, American Chemical Society.

Zinc metal has excellent electrochemical properties of low electrode potential, high specific energy, high specific power, nontoxic, low cost, and natural profusion.^[^ ^46^, ^47^, ^48^ ^]^ Due to the unlimited capacity of the air (as oxygen source), the capacity of ZABs mainly relies on the zinc anode. Therefore, improving the utilization rate of zinc anode is decisive in upgrading the discharge capacity of ZABs. However, the hydrogen evolution corrosion,^[^ ^49^ ^]^ dendrite growth,^[^ ^50^ ^]^ passivation,^[^ ^51^, ^52^ ^]^ and morphological change^[^ ^53^ ^]^ mainly hinder the performance of zinc anode. These inevitable issues are attributed to the inherit characters of zinc anode, such as the spontaneous hydrogen evolution reaction between zinc and electrolyte, which produces hydrogen and causes electrode corrosion, and resultant in the reduced utilization of active substances. In fact, these factors reduce the activity of zinc anode and result in the premature failure of battery. Remarkably, electrospinning technology, as an emerging synthetic method, has a desirable materials compatibility and can be used to solve these problems of zinc anodes. For example, the amphoteric zinc anode is prone to form small dissolution corrosion in alkaline or neutral electrolyte, which eventually causes the corrosion of zinc. In this case, electrospinning can be used to deposit the anticorrosion coating layer on the zinc metal to inhibit the corrosion and hydrogen evolution reaction. Electrospinning and subsequent heat treatment at 350 °C can load a Al_2_O_3_ coating layer on the zinc anode substrate to mitigate the occurrence of corrosion (**Figure** **4**a).^[^ ^54^ ^]^ The rough Al_2_O_3_ layer coating is on the fiber with a diameter of 220 nm (Figure 4b). This Al_2_O_3_ coating shows a good resistance in preventing zinc corrosion (Figure 4c,d). For the hydrogen evolution tests in alkaline solution, the Al_2_O_3_ coating with a thickness of 18 μm shows the best inhibition of hydrogen evolution, where the corrosion current density is 60.6 mA cm^−2^ and the corrosion inhibition efficiency is 88.5%. In the Nyquist plots, the 18 μm Al_2_O_3_ coating exhibits the largest resistance of 5167 Ω cm^2^. It is therefore reasonable to deduce that the thicker coating prevents the zinc anode from corrosion. Meanwhile, the random dissolution and deposition of zinc will also cause the redistribution of active materials on the electrode surface, leading to the morphological change. Eventually, due to the passivation and morphological change, the discharge capacity of ZABs will decrease dramatically, and the cycling life will also be shortened. In this regard, an integrated flexible zinc anode material can be prepared to improve the mechanical properties and avoid corresponding changes. For instance, a heteronanomat (HM) structure of zinc anode is prepared in one step by combining the polyetherimide (PEI) electrospun nanofibers and the zinc powder/single‐walled carbon nanotubes (SWCNTs) hybrid (Figure 4e).^[^ ^55^ ^]^ A porous dimensional structure can be formed to promote the mass transport, which can be observed in the prepared HM zinc anode (Figure 4f). The HM zinc anode can exhibit an outstanding flexibility even after 1000 cycles of bending–twisting tests. For the assembled solid‐state ZAB, a long cycling stability of 1500 min at 10 mA cm^−2^ (10 min per cycle) and good mechanical flexibility can be achieved.

Meanwhile, if the ZnO precipitates and deposits onto the surface of zinc anode, the electrode surface will be blocked and the further reactions will be prohibited. The growth of zinc dendrites is also easily occurred during the operation in alkaline electrolyte. The dendritic growth will eventually pierce the separator, causing the fall off of active materials and failure of the battery. Although there is currently a lack of corresponding reports and research to solve these problems, the corresponding solutions can be proposed based on the mechanism of these reactions and the characteristics of electrospinning. For example, applying a thin electrospun nanofilm contained film‐forming additives, deposition additives, and other materials on the surface of zinc metal was reported to achieve the inhibition of dendritic phenomenon. In addition, other relative researches in the energy storage devices such aqueous zinc ion batteries can also be introduced as the reference for ZABs. As such, by extensively exploring the zinc anode design through anode additives, chemical coating, and structural modifications, the significant progress of the zinc anode can be achieved.

### 3.2 Separators

A separator is essential to prevent the physical contact between the zinc anode and air cathode. An ideal separator should have a high electrical resistance and longstanding tolerance against strong alkaline solution to avoid corrosion and decomposition. Good mechanical properties are also needed for the separator, which can minimize the influence of zinc dendrites and prolong the life span of ZABs.^[^ ^56^, ^57^ ^]^ For the separator in ZABs, it should possess a commendable porosity and ionic selectivity, which not only allows the rapid transportation of hydroxyl ions (OH^−^) but also avoids the migration of the dissolved zincate ions (Zn(OH)_4_^2−^) in the zinc anode region to the air cathode chamber.^[^ ^58^, ^59^ ^]^ The undesirable migration of Zn(OH)_4_^2−^ will poison the air catalysts, and thus reduce the battery performance. Among the massive researches on the fabrication of separators, the characteristics of electrospinning can prepare macrointegrated films, and diversified addition of active materials is very suitable for preparing highly efficient separator in ZABs, endows the long‐stability operation in the device, and provides a chance to develop the advanced batteries, such as the batteries with solid‐state configuration.

**Figure 5:**
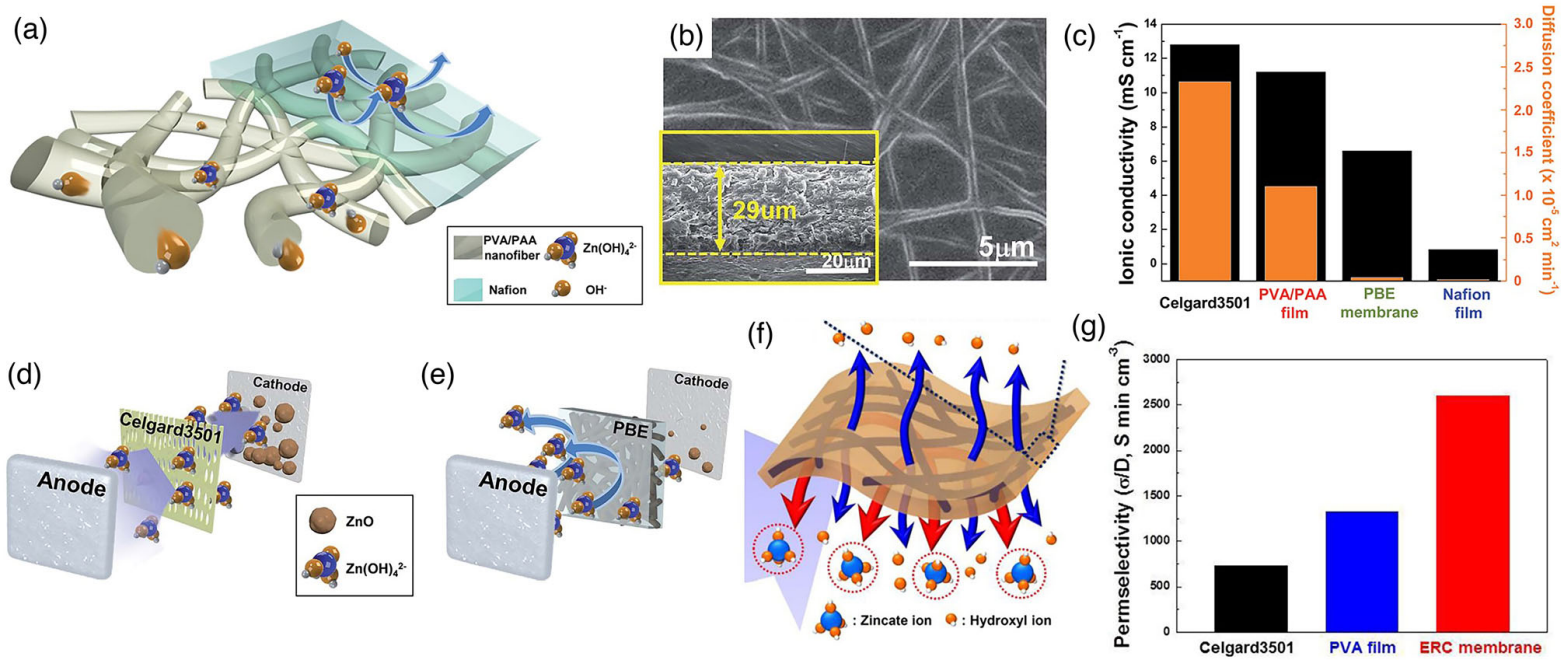
a) Conceptual illustration, b) SEM images, c) ion selectivity curves, d,e) schematic illustration of Zn(OH)_4_^2−^ crossover through the PBE membrane and Celgard 3501.^[^ ^60^ ^]^ Copyright 2016, Royal Society of Chemistry. f) Preparation diagram of ERC membranes and g) comparison of ion selective permeability (σ(OH^−^)/D(Zn(OH)_4_^2−^) of Celgard 3501, PVA film, and ERC membrane. f,g) Reproduced with permission.^[^ ^61^ ^]^ Copyright 2015, Elsevier.

For instance, a separator of polymer blend electrolyte membranes (PBE membranes) with dual‐function of anion conducting and repelling was prepared by the heat treatment of PVA/PAA electrospun nanofibers and the subsequent immersion into Nafion solution (**Figure** **5**a).^[^ ^60^ ^]^ The morphology with negligible aperture in the surface can be exhibited in Figure 5b, which can confirm the intimate combination of Nafion and PVA/PAA nanofibers mat. This structure can provide a basic platform for the great mechanical and electrochemical transfer properties in the assembled ZABs. The PVA/PAA nanofibers mat can play as the anion conducting phase of OH^−^, and the presence of Nafion can function as the anion suppressing phase of Zn(OH)_4_^2−^. The rechargeable ZABs with PBE membranes show an outstanding cycling stability over 2500 min at 20 mA cm^−2^ (10 min per cycle), which is better than the conventional commercial separator of Celgard 3501 (900 min) (Figure 5c). The detailed situation of Zn(OH)_4_^2−^ crossover through PBE membrane and Celgard 3501 is further shown in Figure 5d,e, which can explain the satisfactory performances of PBE‐contained ZABs. In addition to the PVA and PAA, other types of polymers can be combined with electrospinning to prepare the separators. At the same time, an electrospun nanofiber mat‐reinforced perm‐selective composite (ERC) membranes were designed *via* impregnating the polyethyleneimine (PEI) nanofibers mat into PVA (Figure 5f).^[^ ^61^ ^]^ The ERC membranes can exhibit an outstanding ionic perm‐selectivity (σ(OH^−^)/D(Zn(OH)_4_^2−^)) value of 2800 S min cm^−3^ (Figure 5g). It can be attributed to the fact that the PEI nanofibers mat can serve as the skeleton support and improve the stability of active materials, whereas the introduction of PVA matrix can act as the ion selectivity pathways of Zn(OH)_4_^2−^ and OH^−^. Moreover, the mechanical properties are also the main criteria to decide the overall performance of separator. Through the introduction of different components in the hybrid separator, the surrounding performance can be promoted. As such, the prepared PEI nanofibers nanostructure can give a mechanical enhancement in the ERC, which can promote the higher tensile modulus. The synergistic contribution of the internal components endows this separator a great mechanical, thus a satisfactory flexibility can be maintained even in critical environments such as winding and folding situation.

### 3.3 Air Cathode

The discharge and charge process in ZABs suffer from sluggish kinetics of the oxygen electrochemistry, including ORRs/OERs.^[^ ^7^, ^13^ ^]^ The catalytic activity of the oxygen catalyst determines the electrochemical performance of ZABs. Therefore, the designs of cathode electrocatalyst need to follow the principle of high selectivity, long stability, and economical viability during the synthesis. Benefiting from the achievements of electrocatalysts in fuel cells technologies, the oxygen electrocatalyst can be modified by elemental doping, combination with the carbon sheet or metal particles to form the hybrid nanostructure, and can also have a connect with the conductive substrate, these methods can have a significant effect on physical and chemical properties.^[^ ^62^, ^63^, ^64^, ^65^ ^]^ Meanwhile, the other regulation strategies can be used to promote the activity of electrocatalyst, such as the defect engineering, lattice regulation, single atoms design, and introduction of photoelectric field, which can affect the intrinsic activity and have an excellent progress than the traditional catalysts. Moreover, the air electrode also requires the desirable conductivity and porosity to transfer the electrons and reactants. The air cathode itself should have good air permeability, high mechanical properties, and good conductivity, which can infiltrate the catalytic layer without penetration. The characters of electrospinning technology make it demonstrate promising potential in the preparation of electrode architectures integrated with efficient oxygen electrocatalysts and air electrode,^[^ ^44^, ^45^ ^]^ such as the convenient synthesis process, diverse nanostructures, and large‐scale production.

**Figure 6:**
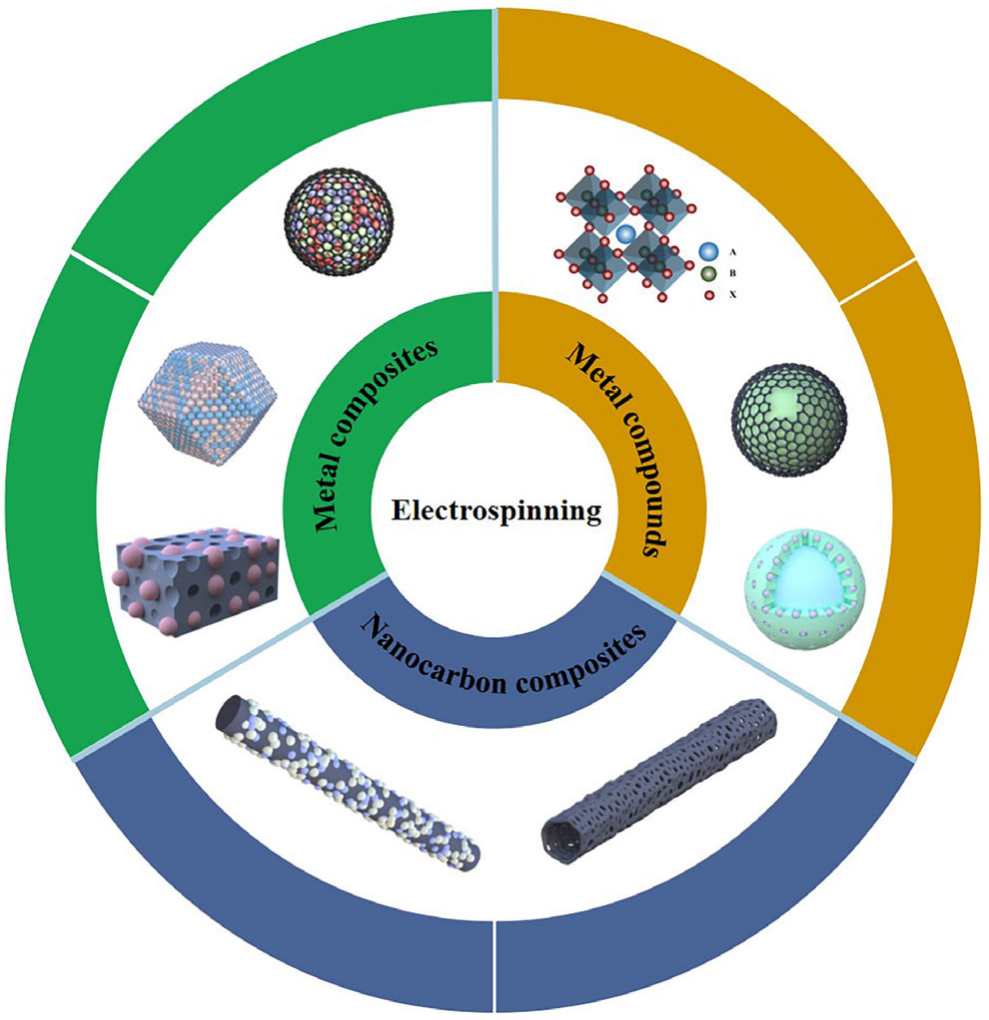
Different categories of oxygen electrocatalysts produced by electrospinning technologies.

In the following content, we will discuss the recent development of oxygen catalysts and free‐standing air electrode achieved by electrospun methods for ZABs. The oxygen catalysts can be mainly divided into nanocarbon composites, metal composites, and metal compounds, as shown in **Figure** **6**.

#### 3.3.1 Nanocarbon Composite Catalyst

**Figure 7:**
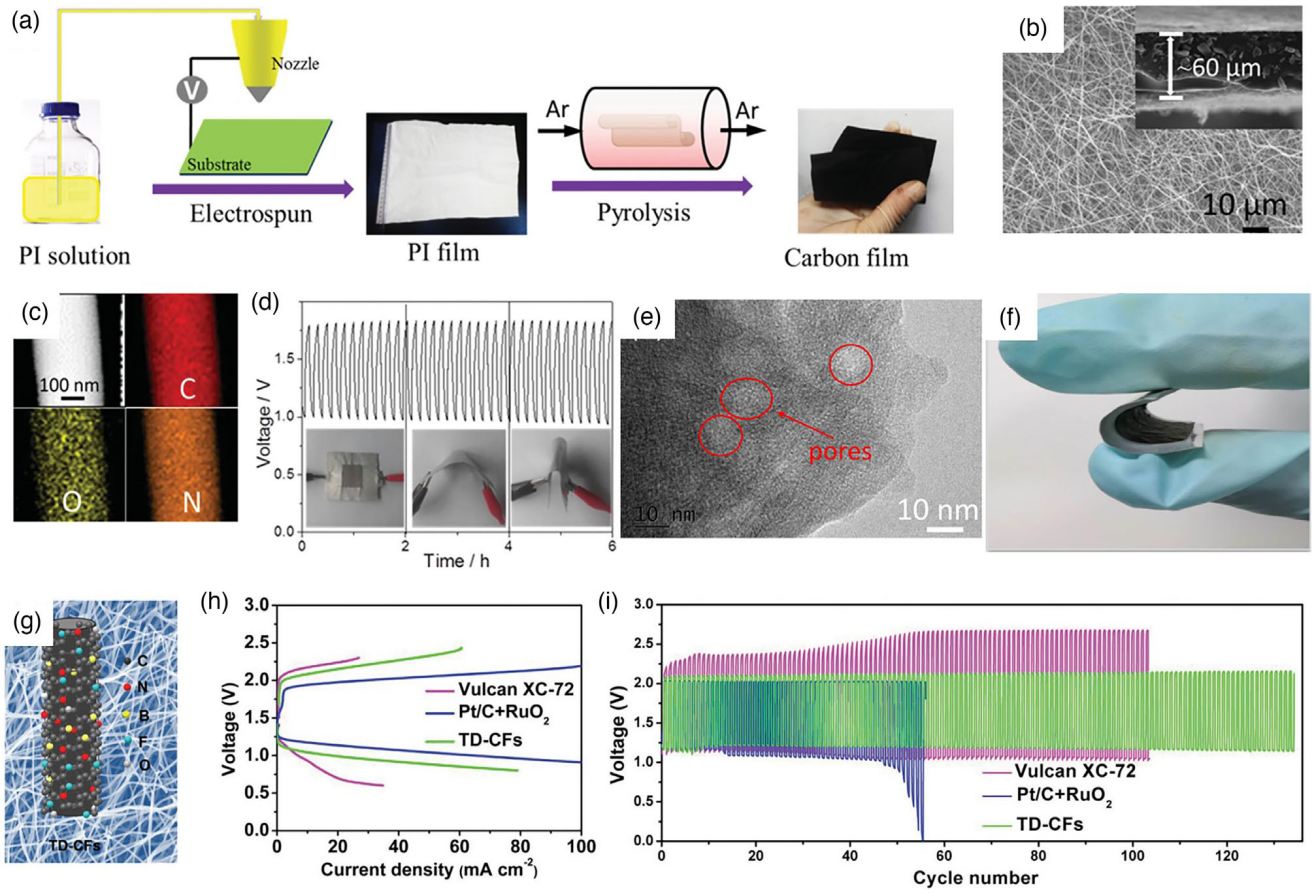
a) Synthetic schematic, b) SEM images, c) elemental mapping, and d) galvanostatic cycling stability of ZABs based on NCNF‐1000 at 2 mA cm^−2^ with different bending conditions. a–d) Reproduced with permission.^[^ ^70^ ^]^ Copyright 2016, Wiley‐VCH. e) High resolution transmission electron microscopy (HRTEM) image and f) photograph of the flexible solid‐state ZAB for NPCNF. e,f) Reproduced with permission.^[^ ^71^ ^]^ Copyright 2019, American Chemical Society. g) Schematic diagram, h) charge/discharge polarization curves, and i) galvanostatic charge–discharge cycling of ZAB using the TD–CFs, Pt/C + RuO_2_, and Vulcan XC‐72 as the cathode. g–i) Reproduced with permission.^[^ ^74^ ^]^ Copyright 2018, Wiley‐VCH.

In recent years, nanocarbon composite catalysts have been extensively studied due to their advantages of large specific surface area, high porosity, good conductivity, and abundant sources.^[^ ^66^, ^67^, ^68^, ^69^ ^]^ Since the electrospinning technology uses carbon‐containing polymers as the spinning precursors, the original unmodified carbon nanofibers can be directly obtained after simple subsequent calcinations. It can be a major means of constructing hybrid carbon nanofibers as the oxygen catalysts to obtain the desired characteristics. The modified electrospun carbon nanofibers can exhibit larger specific surface area and more active sites, which can provide rapid charge transportation and improved electrocatalytic performance. For example, the nanoporous carbon nanofiber films (NCNFs) can be obtained by the calcination of polyimide (PI) electrospun nanofibers (**Figure** **7**a).^[^ ^70^ ^]^ The resultant NCNF‐1000 shows a nanoporous network structure with a large specific surface area up to 1249 m^2^ g^−1^, which provides sufficient active sites and fast electronic conductivity for the catalytic reaction (Figure 7b). Besides, the rich nitrogen and oxygen doping into the NCNF‐1000 results in a good bifunctional performance in oxygen electrocatalysis (Figure 7c). The aqueous ZAB assembled by NCNF‐1000 exhibits a peak power density of 185 mW cm^−2^. Even applied as an air cathode in a flexible solid‐state ZAB, a remarkable cycling stability of 6 h with different bending conditions was obtained (Figure 7d). In addition to the porous structure obtained by calcination, the porous structure can also be formed by introducing foreign materials. Recently, ZnO was introduced in electrospinning to promote the formation of hierarchical porous structure nanofibers (Figure 7e).^[^ ^71^ ^]^ The NPCNFs can be used as a binder‐free self‐standing air cathode in the flexible solid‐state primary ZAB (Figure 7f).

For the internal properties of the electrospun carbon nanofibers, the introduction of nonmetallic heteroatoms (e.g., N, S, O, B, and P) can adjust the electronic properties of carbon matrix and induced defects, which would function as the additional active sites.^[^ ^72^, ^73^ ^]^ For instance, the ternary dopants of N, F, and B can be introduced into the electrospun carbon fibers (TD–CFs) (Figure 7g).^[^ ^74^ ^]^ The TD–CFs exhibit a high activity in ZAB, which was attributed to the modulation of electronic structure and the creation of extra active sites in the carbon nanofibers due to the synergistic effect of doped heteroatoms. The charge/discharge overpotential of TD–CFs is much lower than that of Vulcan XC‐72 (Figure 7h), and a good stability with 120 cycles during 800 min can be obtained as well (Figure 7i).

#### 3.3.2 Metal Composite Catalyst

On the basis of these metal composite catalysts, the catalytic activity can be further improved by introducing metal materials.^[^ ^75^, ^76^, ^77^ ^]^ The metal elements can enhance the graphitization degree of carbon materials during the pyrolysis process, promote the electron transfer, and have a synergetic effect with the carbon nanostructure to from more active sites in the catalysts.

**Figure 8:**
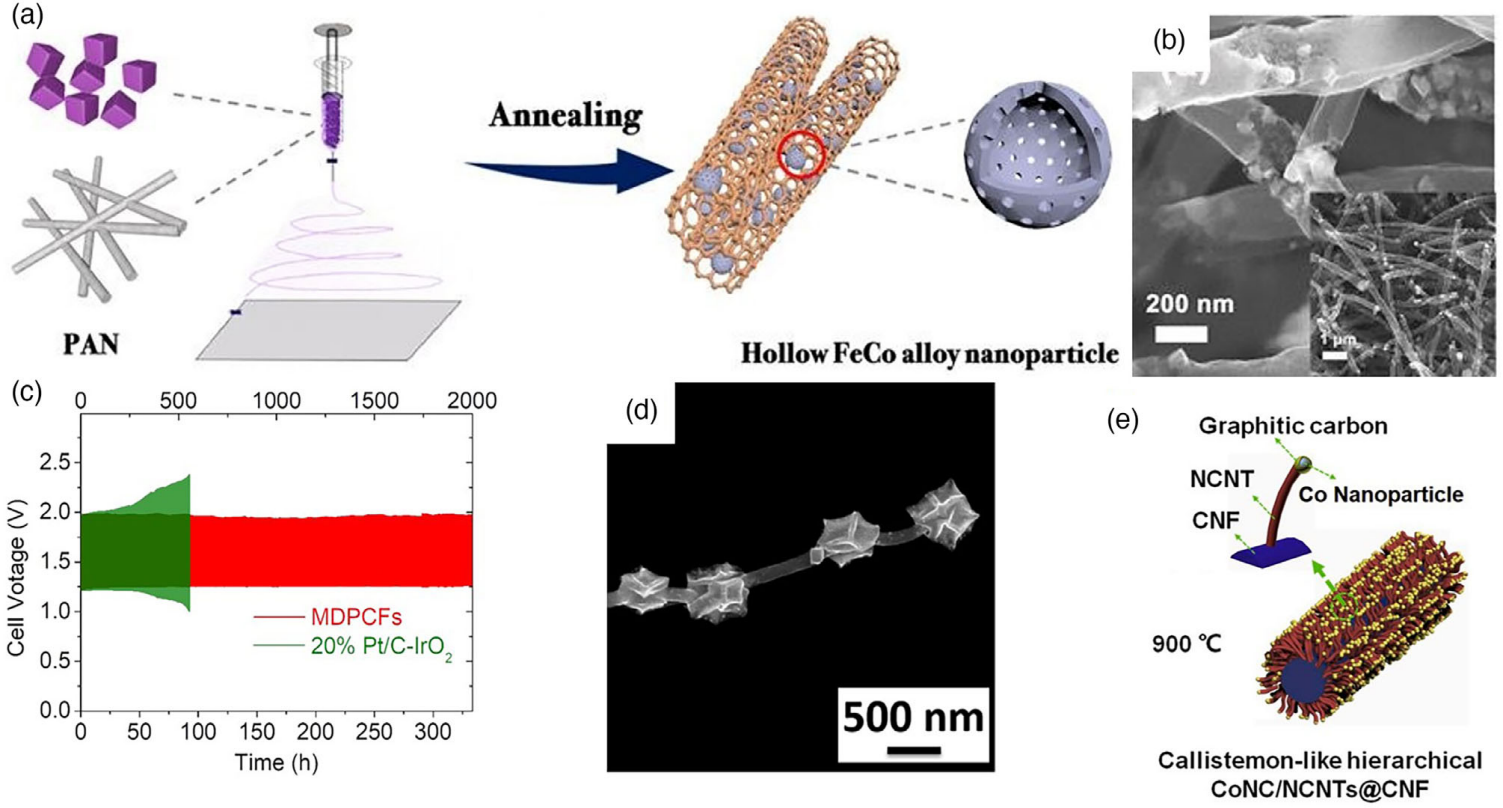
a) Synthesis illustration and b) SEM images of h‐FeCo/N‐CNFs. a,b) Reproduced with permission.^[^ ^84^ ^]^ Copyright 2020, Royal Society of Chemistry. c) Discharging curve and corresponding power density plot of the aqueous ZABs. Reproduced with permission.^[^ ^90^ ^]^ Copyright 2020, Royal Society of Chemistry. d) Field‐emission scanning electron microscopy (FESEM) image of C‐PAN@ZIF‐67. Reproduced with permission.^[^ ^98^ ^]^ Copyright 2019, Elsevier. e) Schematic diagram for the CoNC/NCNTs@CNF. Reproduced with permission.^[^ ^100^ ^]^ Copyright 2020, Elsevier.

Many reports have been devoted to the exploration of integrating metal‐based catalysts in the electrospun carbon nanofibers, which can be attribute to the advantages in low cost, a wide range of potential candidates, and excellent thermal stability of metal catalysts.^[^ ^78^, ^79^, ^80^, ^81^ ^]^ Currently, intensive research efforts are focusing on how to obtain metal‐based catalysts materials by combining polymers and metals through electrospinning and subsequent processing. For instance, the multimetallic particles on the nanofibers can provide more active sites, adjust the electronic state around the carbon atoms, and improve the oxygen catalytic activity.^[^ ^82^, ^83^ ^]^ In a recent study, the hollow FeCo alloy nanoparticles were distributed in the N‐doped carbon nanofibers through the electrospinning and annealing process, as shown in **Figure** **8**a,b.^[^ ^84^ ^]^ The hybrid manifests a large active surface area and abundant defects on the surface. More significantly, an outstanding cycling stability of 600 cycles (200 h) at a current density of 10 mA cm^−2^ in aqueous ZAB is obtained. Except for the bimetallic alloys, ternary alloys with excellent catalytic properties have also been reported for ZABs.^[^ ^85^ ^]^ For instance, mesoporous carbon nanofibers loaded with FeCoNi ternary alloy (FeCoNi–CNF) are prepared through three kinds of metal precursors.^[^ ^86^ ^]^ It can exhibit an small overpotential of 220 mV at a current density of 10 mA cm^−2^ in OER.

In addition to the multielement alloy strategy that improves the catalytic performance by introducing extra metal active sites, the introduction of metals can also interact with other elements to generate the metal–nitrogen–carbon (M–N–C) active sites through the simple pyrolysis process.^[^ ^87^, ^88^, ^89^ ^]^ It exhibits outstanding oxygen catalytic activity through the interaction of metals and nitrogen doping in the carbon nanofibers. The CoFe–glycerate microspheres derived from electrospinning and calcination possessed rich Fe/Co–N–C active sites. It also show the high peak power density of 288.8 mW cm^−2^ and long‐term cycling stability of 2000 cycles at 10 mA cm^−2^ in the aqueous ZAB (Figure 8c).^[^ ^90^ ^]^ The metal‐organic framework (MOF) materials can be used as another ideal precursors to prepare the air catalysts with abundant M–N–C active sites.^[^ ^91^, ^92^, ^93^, ^94^, ^95^, ^96^, ^97^ ^]^ For example, the ZIF‐67 derived Co/N–C particles on the PAN‐based carbon nanofibers (C‐PAN@ZIF‐67) with gems‐on‐string structure and more Co–N–C active sites exposure, which exhibit a long‐term stability of 965 h (*j* = 10 mA cm^−2^, 10 min per cycle) (Figure 8d).^[^ ^98^ ^]^ Similarly, bimetallic Zn/Co–ZIF was used to synthesize the CNF@Zn/CoNC.^[^ ^99^ ^]^ Due to the active sites of N–C, Co–N_*x*_, Co@C species, and the 1D porous conductive carbon nanofibers structure, the ZAB exhibits a good stability of 410 cycles (150 h, *j* = 2 mA cm^−2^). In addition, other activated carbon materials can be further introduced to MOFs. For instance, ZIF‐8/ZIF‐67 is in situ grown on the surface of electrospun PAN nanofibers, after calcination, N‐doped carbon nanotubes can be obtained with graphitic carbon encapsulated the Co nanoparticles (CoNC/NCNTs@CNF) (Figure 8e).^[^ ^100^ ^]^ The introduction of NCNTs can further improve the mass transport and produce Co–N–C active sites in the hybrid. It exhibits a diffusion‐limiting current density of 5.6 mA cm^−2^ for ORR, and a small overpotential of 0.39 V for OER. The aqueous ZAB also shows a high peak power density of 260 mW cm^−2^.

#### 3.3.3 Metal Compound Catalyst

**Figure 9:**
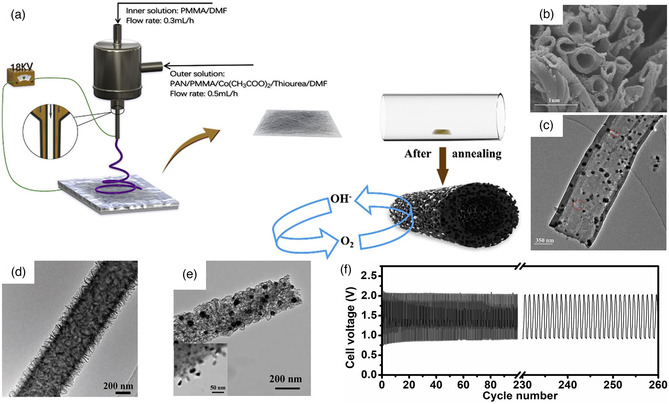
a) Synthesis scheme, b) SEM image, and c) TEM image of Co_9_S_8_‐NSHPCNF. a–c) Reproduced with permission.^[^ ^107^ ^]^ Copyright 2019, Elsevier. d) TEM image of CuCo_2_S_4_NSs@N‐CNFs. Reproduced under the terms of the CC‐BY 4.0 license.^[^ ^108^ ^]^ Copyright 2019, The Authors, published by Wiley‐VCH. e) TEM images of Fe/N‐HCNFs and its f) galvanostatic cycling stability at the density current of 20 mA cm^−2^. e,f) Reproduced with permission.^[^ ^110^ ^]^ Copyright 2019, Wiley‐VCH.

Meanwhile, metal compounds can be obtained by further doping with other nonmetal elements to replace the initial atoms based on the previous catalyst preparation. Diversified types and unique structures can be formed by adjusting the amount and types of nonmetallic materials doped, such as the various metal derivatives of metal phosphides, chalcogenides, and carbides.^[^ ^101^, ^102^, ^103^, ^104^, ^105^, ^106^ ^]^ For instance, the Co_9_S_8_ nanoparticles embedded in the N/S‐doped carbon nanofibers are synthesized by coaxial electrospinning (**Figure** **9**a–c).^[^ ^107^ ^]^ Due to the Co—S covalent bond and Co active sites in Co_9_S_8_, the hollow porous structure of carbon nanofibers and doping of N/S heteroatoms can promote the catalytic performance. The aqueous ZAB exhibits an outstanding cycling stability of 1000 cycles (166 h) at 2 mA cm^−2^. Recently, a composite spinel‐type structural polymetallic sulfide of CuCo_2_S_4_ nanosheets was reported.^[^ ^108^ ^]^ The CuCo_2_S_4_ nanosheets are uniformly distributed on the surface of the N‐doped carbon nanofibers to form the core–shell structure (Figure 9d), where the solid‐state ZAB delivers a large specific capacity of 896 mA h g_Zn_^−1^ under a current density of 25 mA cm^−2^ and the 93.62% capacity retention after the bending test (1000 cycles, from 0° to 180°). In addition, the Co_2_P and CoN_*x*_ moieties are anchored in the N/P‐doped carbon nanofibers by the introduction of phytic acid, which enhance the active sites of Co_2_P@CNF.^[^ ^109^ ^]^ In addition, through the carbonization of PAN/ZIF‐8 electrospun nanofibers, tannic acid (TA) and FeCl_3_ are directly used to prepare the Fe_3_C/Fe nanoparticles supported on hollow porous N‐doped carbon nanofibers (Fe/N–HCNFs).^[^ ^110^ ^]^ The Fe_3_C/Fe nanoparticles have a small size (about 20–50 nm) as shown in Figure 9e, which provide abundant active sites synergized with HCNFs. The assembled ZAB can show a long‐term cycling stability of 260 cycles at 20 mA cm^−2^ (Figure 9f).

**Figure 10:**
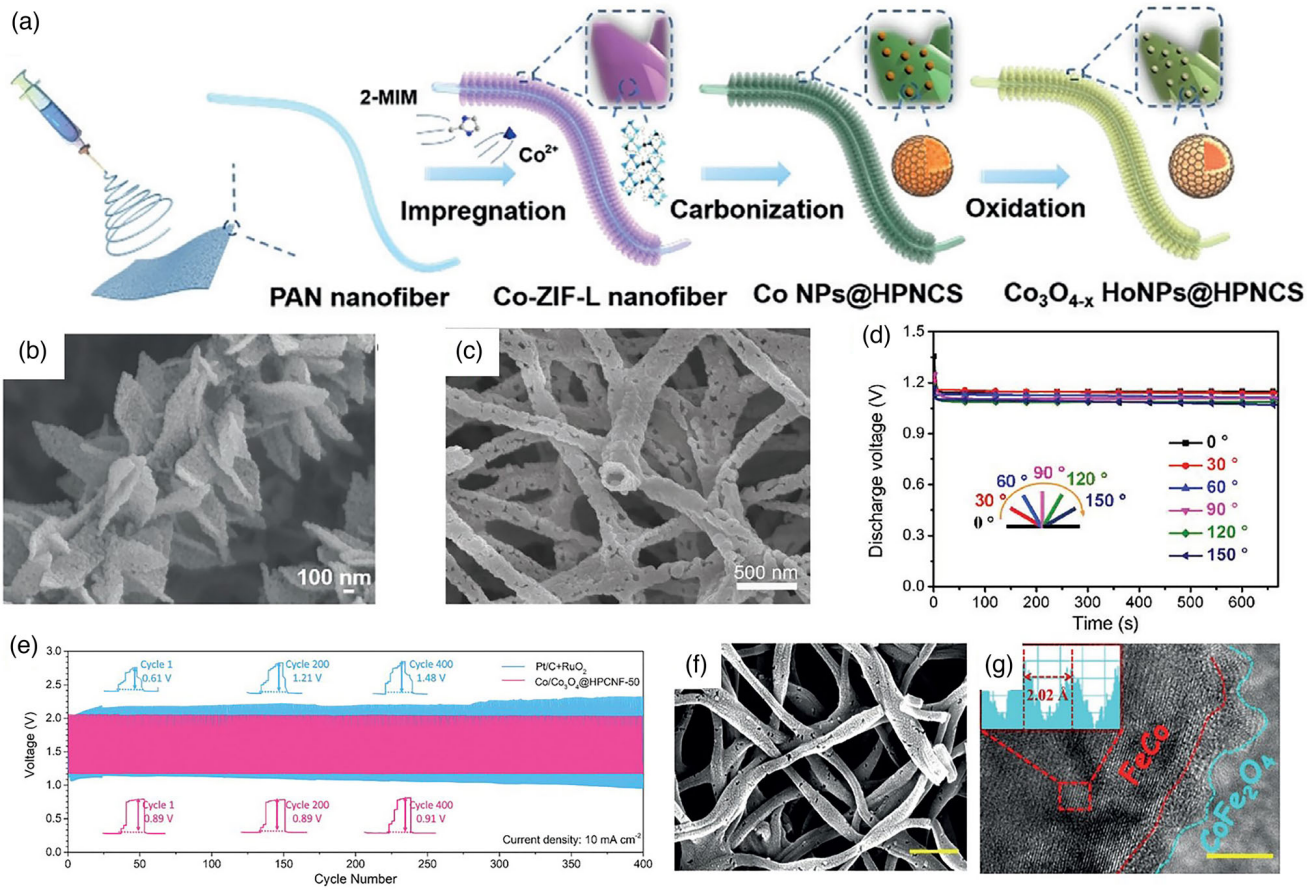
a) Synthetic process and b) SEM image of Co_3_O_4−*x*_ HoNPs. a,b) Reproduced with permission.^[^ ^122^ ^]^ Copyright 2019, Wiley‐VCH. c) SEM image and d) its flexible CMO/S‐based ZABs discharge curves at a current density of 1 mA cm^−2^ with different bending angles. c,d) Reproduced with permission.^[^ ^125^ ^]^ Copyright 2018, Wiley‐VCH. e) Galvanostatic cycling stability of rechargeable ZABs. Reproduced with permission.^[^ ^144^ ^]^ Copyright 2020, Wiley‐VCH. f,g) TEM images of the FeCo and CoFe_2_O_4_. Reproduced with permission.^[^ ^145^ ^]^ Copyright 2020, Wiley‐VCH.

In addition to the aforementioned metal‐derived catalysts, as the most typical type of metal compound material, metal oxides can be obtained by further adjustable oxidizing calcination under air atmosphere. Meanwhile, metal oxide has a better affinity binding effect for oxygen, which can exhibit the character of excellent stability under alkaline or acidic conditions, and further contribute a synergetic effect with the carbon electrospun nanofibers.^[^ ^111^, ^112^, ^113^, ^114^, ^115^, ^116^, ^117^, ^118^, ^119^, ^120^, ^121^ ^]^ Recently, the Co_3_O_4_ hollow particles (Co_3_O_4−*x*_ HoNPs) supported on hierarchically porous N‐doped carbon structure (HPNCS) are prepared by the growth of leaf‐like Co‐ZIFs and subsequent heating treatment of electrospun nanofibers (**Figure** **10**a).^[^ ^122^ ^]^ The carbon flake structure can be distributed on carbon nanofibers, which have a large surface area and rich oxygen vacancy defects (Figure 10b). The Co_3_O_4−*x*_ HoNPs@HPNCS‐60 shows the best ORR (*E*_1/2_ = 0.834 V) and OER (*η*_j=10_ = 313 mV) performance. In addition, there is also the bimetallic compounds with different chemical compositions.^[^ ^123^ ^]^ For instance, coaxial electrospinning technology synthesized N‐doped thin‐walled CuCo_2_O_4_@C nanotubes (CCO@C) with mesoporous structure.^[^ ^124^ ^]^ The mesoporous open‐ended hollow structure can increase the exposure of active sites and enhance the mass transport process. The sulfur‐doped CaMnO_3_ nanotubes (CMO/S) were synthesized *via* sulfur doping (Figure 10c).^[^ ^125^ ^]^ The sulfur heteroatoms in CMO can produce the oxygen vacancies and change the electronic structure, which can effectively enhance the O_2_ adsorption and activation capability. The CMO/S‐300 exhibited an excellent flexibility with different bending angles from 0° to 150° in a solid‐state ZAB (Figure 10d).

The active metal oxides can be combined with the different kinds of metal‐based nanoparticles by controlling the precursor composition and subsequent pyrolysis process, and achieve more excellent catalytic performance.^[^ ^126^, ^127^, ^128^, ^129^, ^130^, ^131^, ^132^, ^133^, ^134^, ^135^, ^136^, ^137^, ^138^, ^139^, ^140^, ^141^, ^142^, ^143^ ^]^ Recently, Co/Co_3_O_4_ hybrid nanoparticles are embedded in the hollow porous carbon nanofibers (Co/Co_3_O_4_@HPCNF).^[^ ^144^ ^]^ Through the porous structure design with different relative humidity during electrospinning process, an outstanding cycling stability with slight voltage changes (0.89–0.91 V after 400 cycles) can be seen in the as‐fabricated ZAB (Figure 10e). Similarly, coaxial electrospinning technology and the subsequent pyrolysis is also used to prepare the bamboo‐shaped fibrous catalysts (Figure 10f).^[^ ^145^ ^]^ The as‐prepared catalysts exhibits the porous structures in which the FeCo alloy and the corresponding CoFe_2_O_4_ are formed at the edge of carbon nanofibers (Figure 10g). The hybrid catalyst manifests a high half‐wave potential of 0.90 V for ORR.

### 3.4 Free‐Standing Air Electrode

Although the development of oxygen electrocatalysts has made great progress, their actual performance in ZABs is still greatly limited. One of the main reasons is the poor integration of catalysts and electrode substrate in the traditional air electrode structure, which limit the original catalytic performance of the catalysts.^[^ ^146^ ^]^ Generally, to achieve the desirable charge transport and product diffusion, the air electrode is manufactured by mixing the oxygen electrocatalyst and auxiliary additives in the construction of the gas diffusion layer.^[^ ^147^, ^148^ ^]^ The common polymer binder and catalyst conductive support are Nafion and acetylene black, respectively. However, these auxiliary materials have a negative effect on battery performance,^[^ ^149^, ^150^ ^]^ such as the carbon corrosion occurred on these auxiliary materials will increase the aggregation/sintering and even dissolution of the catalysts, seriously reducing the overall battery performance. Therefore, it is necessary to design a new integrated self‐standing air cathode without the additional auxiliary carbon and binder as well as the requirement of complicated preparation process.^[^ ^151^, ^152^, ^153^ ^]^ Electrospinning technology can be considered as a convenient method to fabricate the free‐standing air cathode. As such, it can directly prepare the self‐supporting nanofilms without any modification, and the compatibility can allow it to contain many functional nanoparticles as the precursor for the preparation of the next process. Meanwhile, the catalytic materials can have a strong connection with the electrospun nanofiber substrate due to the in situ synthesis method, which can avoid the particles agglomeration and fall off, maintain the long stability and high activity.

**Figure 11:**
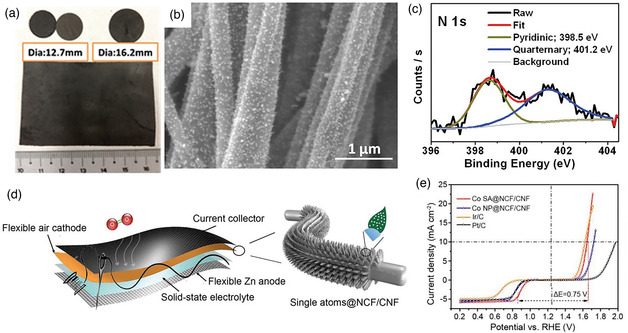
a) Digital photography, b) SEM image, and c) X‐ray photoelectron spectroscopy (XPS) spectrum for C–CoPAN900. a–c) Reproduced with permission.^[^ ^154^ ^]^ Copyright 2015, Royal Society of Chemistry. d) Synthetic route and e) electrochemical activities of Co SA@NCF/CNF. d,e) Reproduced with permission.^[^ ^155^ ^]^ Copyright 2019, Wiley‐VCH.

**Figure 12:**
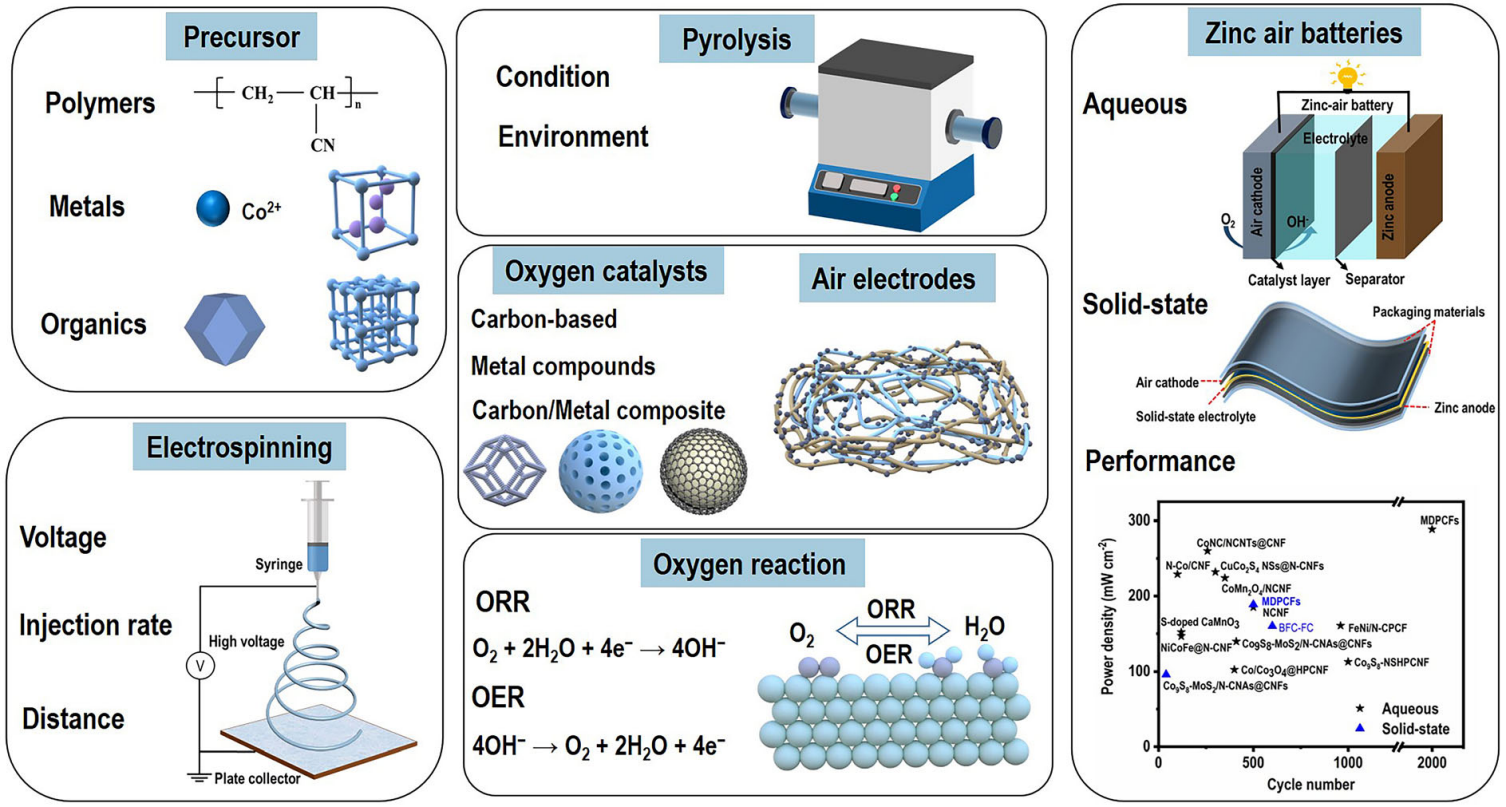
Summary of electrospun nanomaterials for ZABs.

For instance, a Co_3_O_4_‐modified carbon nanofiber (C‐CoPAN) is directly used as a disc or rectangular binder‐free self‐standing air cathode in the aqueous ZAB (**Figure** **11**a).^[^ ^154^ ^]^ The C‐CoPAN900 obtained by pyrolysis at 900 °C has two nitrogen dopants in the form of pyridine and quaternary nitrogen. The 3D architecture in the carbon nanofibers can increase the specific surface area and enhance the mass transport (Figure 11b,c). Meanwhile, when Co single atoms supported by N‐doped carbon flake arrays are grown on carbon nanofibers (Co SA@NCF/CNF), it can be directly applied to flexible solid‐state ZAB as the self‐standing air cathode in the form of thin films, and these characteristics indicate a promising potential of an integrated battery system (Figure 11d).^[^ ^155^ ^]^ The sufficient active sites from Co single atoms and the porous structure together contribute to an excellent catalytic performance of Co SA@NCF/CNF. It shows the highest ORR half‐wave potential (*E*_1/2_) of 0.88 V and a small OER overpotential with 400 mV at the current density of 10 mA cm^−2^ (Figure 11e). The more detailed information for oxygen catalysts and free‐standing electrodes, such as electrospinning preparation parameters, catalyst and electrode types, heating treatments, and the performance of the corresponding ZABs, are listed in **Figure** **12**. Each of them is necessary to get the ideal electrospun nanofiber materials, a small change in them may bring an unexpected result, a system and detailed project should be considered during these sections. There is a list of the overall synthesis and application of electrospinning technology in the ZABs, a clear pathway of the electrospinning in the ZABs can be shown, which hope can provide a guidance for the design, regulation, and assembling of electrospun nanofiber materials in the ZABs.

## 4 Conclusion and Perspective

Due to the high specific energy density, operational stability, and low cost, ZABs have been receiving more and more attention in energy‐related researches. However, their further application still faces several problems, such as sluggish ORR/OER reaction, zinc dendrite, and electrolyte leakage. As a technology for preparing nanofiber materials, electrospinning may provide solutions for the development of ZABs. First, the porous nanofibers can favor sufficient contacts between the active materials/catalysts and electrolytes to shorten the ions diffusion pathways for enhanced rate capability. Second, the high electronic conductivity of these nanofibers can further enhance the reaction activity, accelerating the electrons transport process in ORR/OER. Third, the structural stability of electrospun nanofibers endows the feasible fabrication of self‐supporting electrodes without the addition of adhesives and conductive agents. Moreover, the morphology and structure of electrospun nanofibers can also be further ever‐changing for functionalized nanofiber hybrids, which can achieve the diverse functions and guide the design of materials for the expected performance.

In this review, the basic electrospinning technology and its association with other methods (such as hydrothermal and calcination treatments) have been introduced as an efficient and versatile approach to enable the fabrication of hybrid nanofiber materials with various architectures and compositions for high‐efficiency ZABs. Despite the developments of electrospinning technology for advanced porous nanomaterials applied in ZABs have achieved great progress in recent years, the strategies involving catalysts/electrodes synthesis, electrocatalytic characterization, and device assembly are complex interdisciplinary systems, where several significant problems and challenges still remain, requiring further discussion and innovative techniques for upgradation. 1) Electrospinning technology: most of developed electrospinning technologies applied in ZABs mainly focus on the synthesis of air catalysts/cathodes. Only a few reports stress on the zinc anodes and separators, which may face some great challenges such as corrosion and zinc dendrites. The structural designs of electrospun materials are mostly confined to 3D network‐like porous or hollow structures, which can be easily obtained by high‐temperature calcination. However, this does not give full utilization to the superiority of electrospinning technology, which can produce nanofibers with diverse structures, such as helical and pod‐shaped fibers by the special roll–roll devices or microfluidic electrospinning process. Meanwhile, the polymer source in electrospinning technology is commonly polyacrylonitrile, which could not fully meet the rapid development of ZABs due to the simple elemental component of C, N, and O. If require the materials have the ability of hydrophilic, hydrophobic, acid‐resistant, or alkaline‐resistant, the polymer need to be in contact with other materials, such as metals, and carry out subsequent modification steps such as further calcination treatment. 2) Zinc anode: the issues of zinc dendrites, passivation, corrosion, etc. in the deterioration of zinc anodes could not be neglected in ZABs. The zinc anodes those can be used in flexible solid‐state rechargeable ZABs are severely insufficient. Even in the laboratory, the zinc anodes for the experimental tests are unstable, which cannot perform for the long‐term service. The electrospinning technology can be considered to get the modified and even the in situ integrated zinc anodes, which can overcome the dendrites and improve efficiency of zinc. 3) Separator: the highly selective permeability of hydroxide ions and the long‐term stability are the main challenges for the separator membranes. Electrospun nanofiber materials can produce macroscopically visible membranes that can be used in separators. The diversity and controllability of these membrane structures enable them to purposefully solve a series of problems, such as the combination of various polymers and metal ions, organic compounds. 4) Air cathode: these issues in air cathode are the traditional challenges in ZABs, such as the penetration and leakage of electrolytes, carbonation, and corrosion. Thus, the advanced air cathodes with fast mass transfer, favorable oxygen diffusion, high catalytic activity, and stable service performance are expected. It can be designed and fabricated by electrospinning technologies associated with reasonable choose of calcination treatments and active materials. For example, the proper porous carbon nanofibers are electrospun and subsequently calcinated to hybridize with catalyst materials or expose abundant active sites for high‐performance air cathodes. Various electrospun nanofibers‐based self‐supporting binder‐free 3D network structures possess the advantages of low contact resistance, good mechanical properties, and efficient electrolyte diffusion. 5) Electrolyte: the decent electrolytes can improve the mass transfer in the battery and avoid short circuit caused by zinc dendrite. However, there are few studies on the electrolytes (such as electrospun nanofibers based solid‐state electrolytes) at the present stage, which is undeniable that there will be more relevant researches in the future. For instance, it is desirable to form the gel or all solid‐state electrolyte with the combination of traditional liquid electrolytes and electrospun nanofibers, which can expand the application range of ZABs. The other similar energy storage devices that use the electrolyte can also be selected to as the reference direction, including the Li‐ion batteries, Zn‐ion batteries, and fuel cell. Meanwhile, the electrolyte also has the similar work in these devices, such as the addition of organic modified materials can enhance the ion transfer and the modification of the surface of anode and cathode electrodes will prevent the metal dendritic to form the battery short circuit, which can as provide constructive suggestion for the further development. 6) Others: in addition to the several parts of the aforementioned main applications, there are some other functional materials that can be designed to improve related performance in the ZAB. For example, it is necessary to prevent the entry of CO_2_ in ZABs. For the ZAB using alkaline electrolytes, the CO_2_ in the air will diffuse into the battery,^[^ ^156^, ^157^ ^]^ which can carbonize the electrolyte, decreasing the conductivity of the electrolyte, and increasing the internal resistance of batteries. Recently, a carbon dioxide absorption film was used to prevent carbon dioxide from entering the battery system through the vacuum dried PS/PEI electrospun nanofibers membrane.^[^ ^158^ ^]^ PS functioned as a support in the substrate, and PEI can chemically adsorb the carbon dioxide.

**Figure 13:**
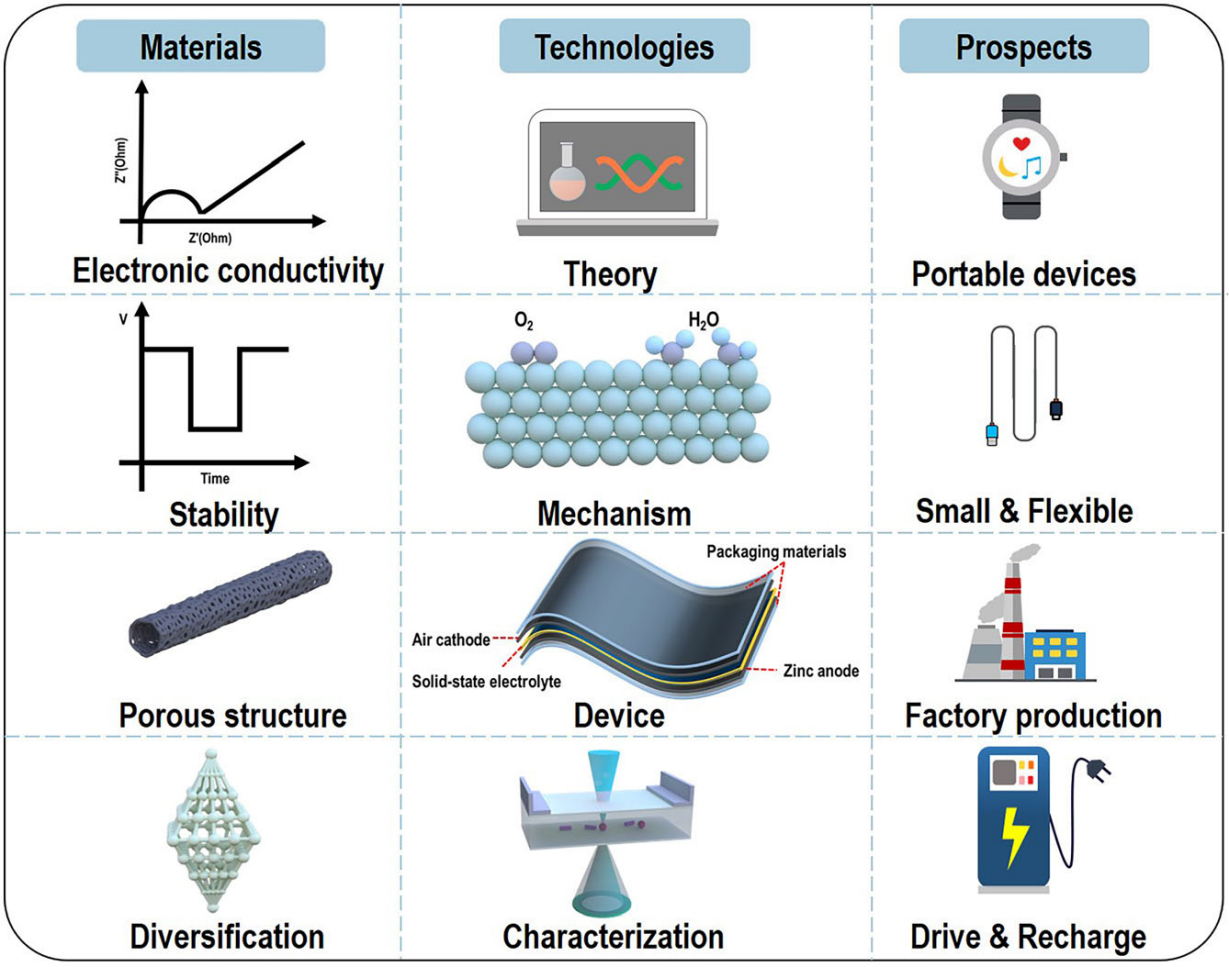
Development perspectives on electrospinning technologies for ZABs.

Although these challenges exist, electrospinning technology has been proved to be an effective strategy for fabricating porous nanofiber materials as different components of zinc anodes, separators, and air catalysts/cathodes in ZABs. The most fundamental starting point for various excellent nanomaterials designed and prepared by electrospinning technology is that the preparation process is facile and fast, which can eventually be mass produced for industrialized large‐scale production applications. With the advances in electrospinning technology and ZABs, the promising prospects are worth being expected as follows. **Figure** **13** exhibits a system prospect of the development of ZABs with electrospun nanofibers as the active materials, and it can be divided into materials, technologies, and prospect of the application. The desirable properties of materials are the main concerned that can exhibit the functional activity at the moment, and the technologies part includes the internal characterization design as well as specific reaction mechanism exploration and final device application, it is the key to understand and apply the prepared materials. On one hand, the rapid development of internet of things, supercomputers, artificial intelligence, and diversified characterization methods can be utilized to exhibit the specific theory and mechanism with a scientific and systematic preparation process, where more novel materials with customized morphologies and structures can be achieved by electrospinning technology. On the other hand, except for zinc anodes, separators, and air cathodes, the packaging materials with satisfactory mechanical properties, and even the nanofiber solid‐state electrolytes with high ionic conductivity for flexible solid‐state ZABs can also be prepared by electrospinning technology. Moreover, the prospect of practical application in the daily life is the ultimate goal to be achieved, and the future applications of electrospun nanofiber materials in ZABs can be divided into two main aspects: small integration and large scale, respectively. The small integration can realize various smart portable devices, such as the fiber‐shaped rechargeable ZABs woven into clothing. The large‐scale application is in the form of large battery stacks, where the high‐performance ZABs stack can be designed as the energy storage and conversion devices with high energy density, small volume and weight, low cost, and high safety for new energy vehicles and factory production equipment. Electrospinning technologies are developing increasingly, which would also accelerate the researches and application of ZABs. Based on these series of discussions, we hope this concise review can provide some useful understandings for electrospinning technologies for ZABs and beyond.

## Conflict of Interest

The authors declare no conflict of interest.
